# Adhesion to oviduct glycans regulates porcine sperm Ca^2+^ influx and viability

**DOI:** 10.1371/journal.pone.0237666

**Published:** 2020-08-21

**Authors:** Sergio A. Machado, Momal Sharif, Govindasamy Kadirvel, Nicolai Bovin, David J. Miller

**Affiliations:** 1 Department of Animal Sciences, University of Illinois at Urbana-Champaign, Urbana, IL, United States of America; 2 Shemyakin Institute of Bioorganic Chemistry RAS, Moscow, Russia; University of Hawai'i at Manoa, UNITED STATES

## Abstract

Before fertilization, sperm bind to epithelial cells of the oviduct isthmus to form a reservoir that regulates sperm viability and capacitation. The sperm reservoir maintains optimum fertility in species, like swine, in which semen deposition and ovulation may not be well synchronized. We demonstrated previously that porcine sperm bind to two oviductal glycan motifs, a biantennary 6-sialylated *N*-acetyllactosamine (bi-SiaLN) oligosaccharide and 3-O-sulfated Lewis X trisaccharide (suLe^X^). Here, we assessed the ability of these glycans to regulate sperm Ca^2+^ influx, capacitation and affect sperm lifespan. After 24 h, the viability of sperm bound to immobilized bi-SiaLN and suLe^X^ was higher (46% and 41% respectively) compared to viability of free-swimming sperm (10–12%). Ca^2+^ is a central regulator of sperm function so we assessed whether oviduct glycans could affect the Ca^2+^ influx that occurs during capacitation. Using a fluorescent intracellular Ca^2+^ probe, we observed that both oviduct glycans suppressed the Ca^2+^ increase that occurs during capacitation. Thus, specific oviduct glycans can regulate intracellular Ca^2+^. Because the increase in intracellular Ca^2+^ was suppressed by oviduct glycans, we examined whether glycans affected capacitation, as determined by protein tyrosine phosphorylation and the ability to undergo a Ca^2+^ ionophore-induced acrosome reaction. We found no discernable suppression of capacitation in sperm bound to oviduct glycans. We also detected no effect of oviduct glycans on sperm motility during capacitation. In summary, Le^X^ and bi-SiaLN glycan motifs found on oviduct oligosaccharides suppress the Ca^2+^ influx that occurs during capacitation and extend sperm lifespan but do not affect sperm capacitation or motility.

## Introduction

In a variety of mammals [1–7], birds, reptiles and amphibians [8–10], after mating sperm are stored in a portion of the female reproductive tract, called the sperm reservoir. The functional sperm reservoir in the mammalian lower oviduct, known as the isthmus, regulates sperm function and extends cell viability, traits necessary for high fertility in species like swine in which semen deposition and ovulation are not always well synchronized [11–13]. Upon semen deposition into the female tract, a sperm subpopulation is transported to the isthmus, where the sperm attaches to epithelial cells until unidentified cues trigger their gradual release towards the ampulla, the fertilization site [2,6]. Sperm binding to epithelial cells regulates sperm function by suppressing sperm motility and prolonging sperm lifespan [14,15]. Sperm binding to the oviduct is believed to be mediated by oviduct carbohydrates [6,16] and the particular adhesion molecules involved in the formation of the sperm reservoir may be species-specific [6,17–19]. The binding of lectin-like receptors on the sperm head to isthmic cell glycans regulates the succession of changes necessary for fertilization, collectively known as sperm capacitation [13,20,21].

During capacitation, plasma membrane potential and ionic transport are altered [22–25], protein phosphorylation is modified [26,27], and there is an efflux of plasma membrane cholesterol [28–30]. Intracellular free Ca^2+^ is central to sperm function in preparation for fertilization [23,25,31,32]. Ca^2+^ influx is necessary for sperm to hyperactivate and ascend beyond the oviduct isthmus to fertilize eggs; failure results in infertility in mice [33,34]. Along with HCO_3_^-^, Ca^2+^ can activate in sperm a soluble adenylyl cyclase (sAC) [35]. The product of sAC, cyclic AMP, activates protein kinase A leading to phosphorylation of a series of proteins [36], although there is also evidence that Ca^2+^ influx occurs after activation of the protein kinase A pathway [37]. It has been proposed that the maintenance of low intracellular Ca^2+^ during the period of adherence to the oviduct epithelium delays capacitation and extends viability, but the mechanism by which this is accomplished is unclear [20,38–40] Regardless, it is clear that the behavior of sperm in the oviduct is heavily dependent on Ca^2+^.

The glycan components that bind sperm and may be responsible for regulating sperm behavior in the oviduct are not completely clear. Although there is considerable evidence that glycans function in cell adhesion [41], it is not known if glycan-mediated adhesion regulates cell function. A glycan array screening of hundreds of specific glycans indicated that all glycans that bound porcine sperm with high affinity contained two motifs, either a biantennary 6-sialylated *N*-acetyllactosamine (bi-SiaLN) structure or a Lewis X trisaccharide (Le^X^) [42,43]. Because these sugars are at least partially involved in sperm binding to porcine isthmic epithelial cells, we hypothesized that oviduct glycoproteins regulate sperm Ca^2+^ influx, motility, acrosome reaction and life span.

## Materials and methods

### Collection and processing of sperm

Several media were used for these experiments. The medium used when sperm capacitation was desired was dmTALP (2.1 mM CaCl2, 3.1 mM KCl, 1.5 mM MgCl2, 100 mM NaCl, 0.29 mM KH2PO4, 0.36% lactic acid, 26 mM NaHCO3, 0.6% BSA, 1 mM pyruvic acid, 20 mM HEPES pH 7.3, 10 U/ml penicillin, 10 μg/ml streptomycin) as described [43]. The medium used when capacitation was not desired lacked BSA and NaHCO3 and was NC-TALP (2.1 mM CaCl_2_, 3.1 mM KCl, 1.5 mM MgCl_2_, 100 mM NaCl, 0.29 mM KH_2_PO_4_, 0.36% lactic acid, 0.6% polyvinyl alcohol, 1 mM pyruvic acid, 35 mM HEPES, [pH 7.3], sterile filtered) as described [44].

### Collection and processing of sperm

For each replicate, semen was collected by applying pressure to the glans penis from 3 to 5 mature *Sus scrofa* boars (Prairie State Semen, Inc., Champaign, IL). Approval from the Institutional Animal Care and Use Committee was not necessary because semen was obtained from a commercial facility. Semen was extended in BSA-free Preserv Xtra (Reproquest, Fitchburg, WI), cooled to 17°C, transported to the laboratory, and processed within 24 h. The extended semen was pooled and 3 ml were washed through a Percoll cushion containing 4 ml of NC-TALP, 0.6 ml of 10X HBS (1.3 M NaCl, 40 mM KCL, 10 mM CaCl_2_, 5mM MgCl_2_, 140 mM fructose, 5% BSA, sterile filtered), and 5.4 ml of Percoll for 10 min at 800 x *g*. The resulting pellet was re-suspended in 5 ml of NC-TALP and centrifuged for 3 min at 600 x *g*. Sperm concentration was estimated by hemocytometer and only samples with greater than 80% motile sperm were used for experiments.

### Sperm binding to glycan coupled to beads and viability assay

Glycan-coated streptavidin-Sepharose High-Performance beads (GE Healthcare Bio-Sciences, Pittsburgh, PA, an average diameter of 34 μm) were used to test the ability of bi-SiaLN and Le^X^ glycan residues to extend the lifespan of non-capacitated porcine sperm. To link glycans to beads, approximately 60 μg of glycans [45] covalently attached to a biotinylated polyacrylamide core were incubated with 20 μl of streptavidin-Sepharose beads for 90 min at room temperature. Each 30-kDa molecule of polyacrylamide had 20% glycan and 5% biotin, by molarity.

To prepare fibronectin-coated beads, fibronectin (FN, Sigma-Aldrich, St. Louis, MO) was first biotinylated by incubating 45 μl of 10 mM biotin with 1 ml of a 1 mg/ml solution of FN, both in PBS, pH 7.35. After incubation for 2 h at 5°C, free biotin was removed using a desalting spin column (2 ml Zeba Spin Desalting Column, 7K MW Cutoff, Thermo Scientific). The biotinylated FN (60 μg) was incubated with 20 μl of streptavidin-Sepharose beads for 90 min at room temperature as above for biotinylated glycans. Beads incubated with biotinylated FN or glycans were washed twice in dmTALP and re-suspended in 100 μl of dmTALP. Once the glycan-coupled beads were ready for use, a 100 μl-droplet containing 2 x 10^5^ sperm/ml was prepared to receive 2 μl of glycan-coated beads. Non-capacitated sperm and beads were co-incubated at 39°C and collected for evaluation of viability at 0.5, 4, 8, 12, and 24 h.

The viability of bound sperm was determined using the LIVE/DEAD Sperm Viability Kit (Life Technologies, Grand Island, NY). Live-cell stain SYBR14 at 100 nM and dead-cell stain propidium iodide at 12 μM were incubated with sperm for 5 min at 39°C. Subsequently, sperm were observed by fluorescence microscopy using a 20X dry objective on a Zeiss Axioskop equipped with an Axiocam (Carl Zeiss, Thornwood, NY) using Zeiss filters 09 (Band pass excitation BP 450–490 nm, beamsplitter FT 510 nm, emission LP 515) for SYBR14 and filter 15 (excitation BP 546/12, beamsplitter 580, emission LP 590) for PI. For each treatment, 10 beads were randomly selected in triplicate droplets and the total number of bound live and dead sperm was enumerated. Some sperm aggregated together and could not be evaluated. For each experiment, sperm bound to 10 beads in triplicate droplets were counted (triplicate droplets in one experiment). So the total number of sperm evaluated in each replicated experiment ranged from about 30 bound to the *N*-acetyllactosamine and suLe^A^ beads to about 200 bound to the suLe^X^ and bi-SialLN beads. At least 100 free sperm for each treatment replicate were also counted. Sperm that were self-agglutinated were not included in the counts. The experiment was documented using AxioVision 4.5 software (Zeiss, Thornwood, NY).

### Measurement of free Ca^2+^ influx in sperm populations

Intracellular Ca^2+^ in sperm populations at a final concentration of 5 x 10^6^ sperm/ml was assessed by a spectrofluorometric assay using the Ca^2+^ probe Fluo-4 as used before [46,47]. Fluo-4 AM was loaded into sperm for 30 min at room temperature at a final concentration of 4 μM and protected from light. After loading, sperm were treated with 40 μg soluble glycans covalently attached to a 30-kDa polyacrylamide chain or the same volume of vehicle control (dmKRBT) were incubated at 39°C and measurements were taken on aliquots every 30 min for 90 min after glycan addition. To account for probe leaking and extrusion from cells, 8.4 mM EGTA was used to chelate extracellular Ca^2+^ just before each measurement. Some samples were treated with 5 μM ionomycin as a positive control [48]. Ca^2+^ binding to Fluo-4 was detected by argon-ion laser excitation at 494 nm and emission at 516 nm in a QuantaMaster 4CW fluorescence spectrophotometer (Photo Technology International, North Edison, NJ).

### Assessing capacitation by evaluating sperm motility patterns

**Fig 1:**
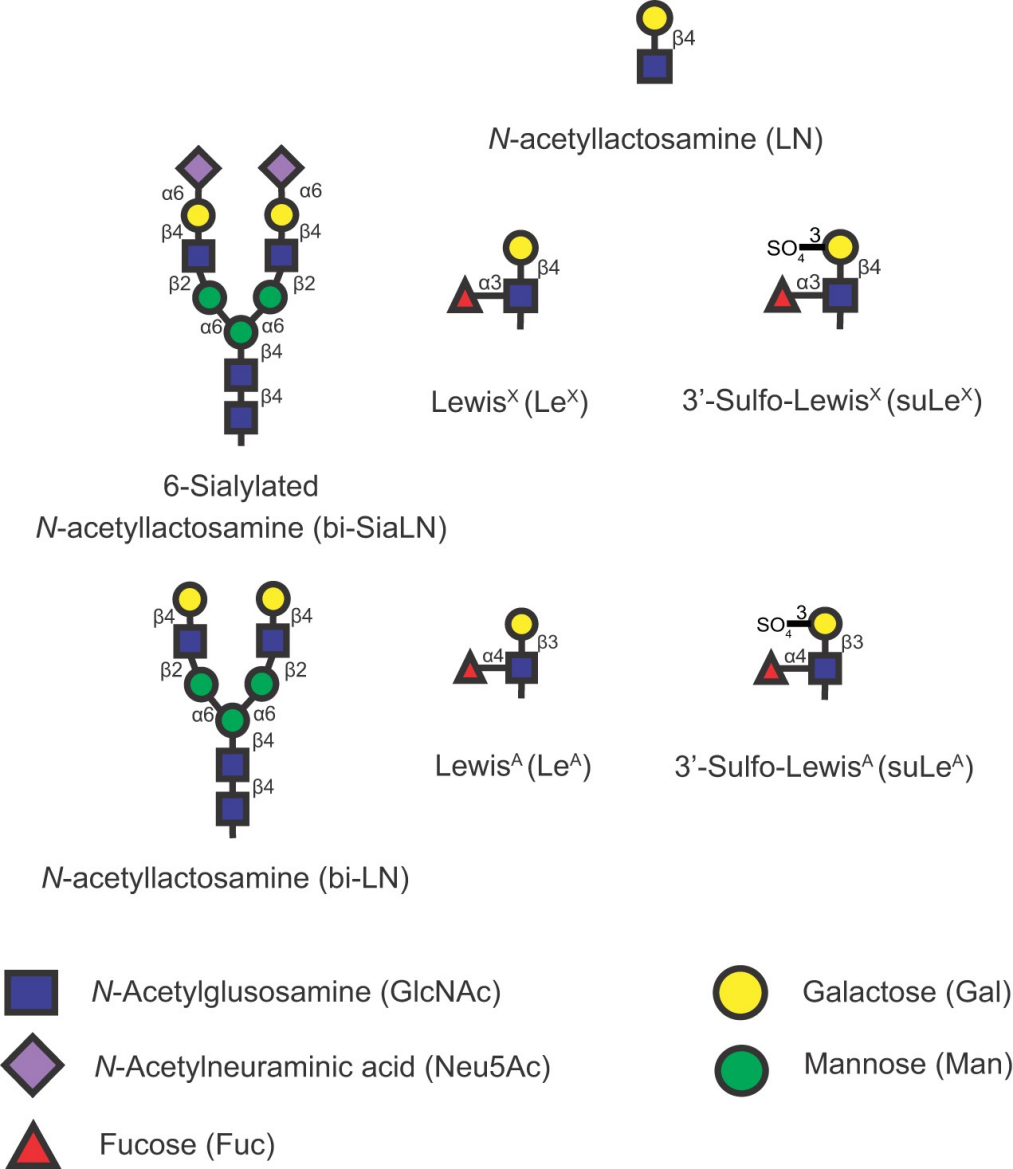
Structures of glycans used. The glycans used in this study are presented showing composition and linkages. The symbols that represent each monosaccharide are shown at the bottom of the figure. bi-SiaLN and Le^X^ structures are found in the oviduct. Lewis A trisaccharide is an isomer of Le^X^ but does not bind porcine sperm and was used to assess non-specific effects. The disaccharide *N*-acetyllactosamine is a component of both bi-SiaLN and Lewis structures but it also does not bind sperm and was also used to measure non-specific effects.

Motility of sperm bound to soluble glycans was assessed using the Hamilton Thorne Semen Analysis CASA system (Hamilton Thorne, Beverly, MA, USA). Sperm were incubated with 40 μg of each soluble glycan (bi-SiaLN, suLe^X^, LN and suLe^A^; Fig 1) attached to a 30 kDa polyacrylamide chain at 39°C in normal dmTALP and NC-TALP or in dmTALP or NC-TALP without glycans as a control for 4 h. Hyperactivation was assessed by examining curvilinear velocity, linearity, and amplitude of lateral head displacement [49]. For each experimental condition, 5 random fields were evaluated for a minimum total of 100 cells (in each field) in replicates.

### Assessing capacitation by the ability to undergo an induced acrosome reaction

Capacitation status of sperm incubated with soluble oviduct glycans (bi-SiaLN, suLe^X^, LN and suLe^A^) was assessed by the ability to undergo a Ca^2+^ ionophore (A23187)-induced acrosome reaction. Sperm were incubated with 40 μg of soluble glycans per ml at 39°C in either dmTALP or NC-TALP. Acrosome status was assessed on aliquots at 0, 2 and 4 h of incubation after addition of 5 or 10 μM A23187 and, 10 min later, Coomassie staining [50]. Briefly, sperm were fixed, attached to microscope slides, and stained with Coomassie Blue G-250. A minimum of 200 sperm were examined for each treatment in each replicate.

### Assessing capacitation by sperm protein tyrosine phosphorylation

Changes in sperm protein tyrosine phosphorylation during capacitation were assessed using SDS-PAGE and immunoblotting. After washing, sperm concentration was adjusted to 5 x 10^6^ cells for each treatment and sperm were incubated in NC-TALP (negative control for tyrosine phosphorylation), dmTALP (positive control for tyrosine phosphorylation), and dmTALP containing 40 μg/ml of either bi-SiaLN, suLe^X^, Le^X^, LN, suLe^A^ or Le^A^ (Fig 1). Sperm were incubated at 39°C with the soluble oviduct glycans and aliquots were collected at 0, 2 and 4 h. At each time point, aliquots were centrifuged at 13,000 x g for 5 min at 4°C. The supernatant was discarded, and the pellet was re-suspended in ice-cold Nonidet-P40 Lysis Buffer (150 mM NaCl, 50 mM Tris, pH 8.0, and 1% NP-40) containing 0.2 μM sodium orthovanadate, in addition to a protease inhibitor cocktail containing AEBSF, bestatin, E-64, pepstatin A, phosphoramidon, and leupeptin (Millipore-Sigma, St. Louis, MO). After homogenization by repeated pipetting, the samples were boiled for 5 min and centrifuged at 13,000 x g for 5 min at 4°C. The resulting supernatant was transferred to a fresh micro-centrifuge tube containing 5% β-mercaptoethanol (final concentration) and boiled 5 min. Aliquots containing 5 x 10^6^ sperm were diluted in 5X loading buffer (4% SDS, 20% glycerol, 0.1% bromophenol blue, 0.125 mM Tris HCl, pH 6.8), and loaded into a 4–20% gradient gel (Thermo Fisher Scientific Inc., Waltham, MA). After electrophoresis, proteins were transferred to a nitrocellulose membrane. The membranes were blocked with 5% BSA and incubated with primary antibody. Phosphotyrosine antibody (4G10, Millipore-Sigma, St. Louis, MO) was used at 1:1000 dilution in TBST (20 mM Tris, 150 mM NaCl, 0.1% Tween 20) with 5% BSA. Membranes were washed in TBST and incubated with a polyclonal anti-mouse IgG conjugated to HRP (BD Pharmingen, San Jose, CA) diluted 1:2000. After washing, the membranes were incubated with a chemiluminescent peroxidase substrate (Thermo Fisher Scientific Inc., Waltham, MA). Chemiluminescent signals were documented using an ImageQuant LAS 4000 (GE Healthcare Bio-Sciences, Pittsburgh, PA). The primary antibody was replaced with normal IgG as a control. Three independent biological replicates were done for each treatment and time point.

### Statistical analysis

Replicates were performed independently using semen pooled from different boars. At least 3 replicates were performed for each condition. Differences among means were determined using a one-way analysis of variance in SAS (v. 9.1 SAS Institute, Inc, Cary, NC). The results are shown as means ± SEM and the means were considered to belong to distinct populations if *P* < 0.05 using Tukey’s test for multiple comparisons.

## Results

### Binding to bi-SiaLN and Le^X^ glycans enhances sperm viability

**Fig 2:**
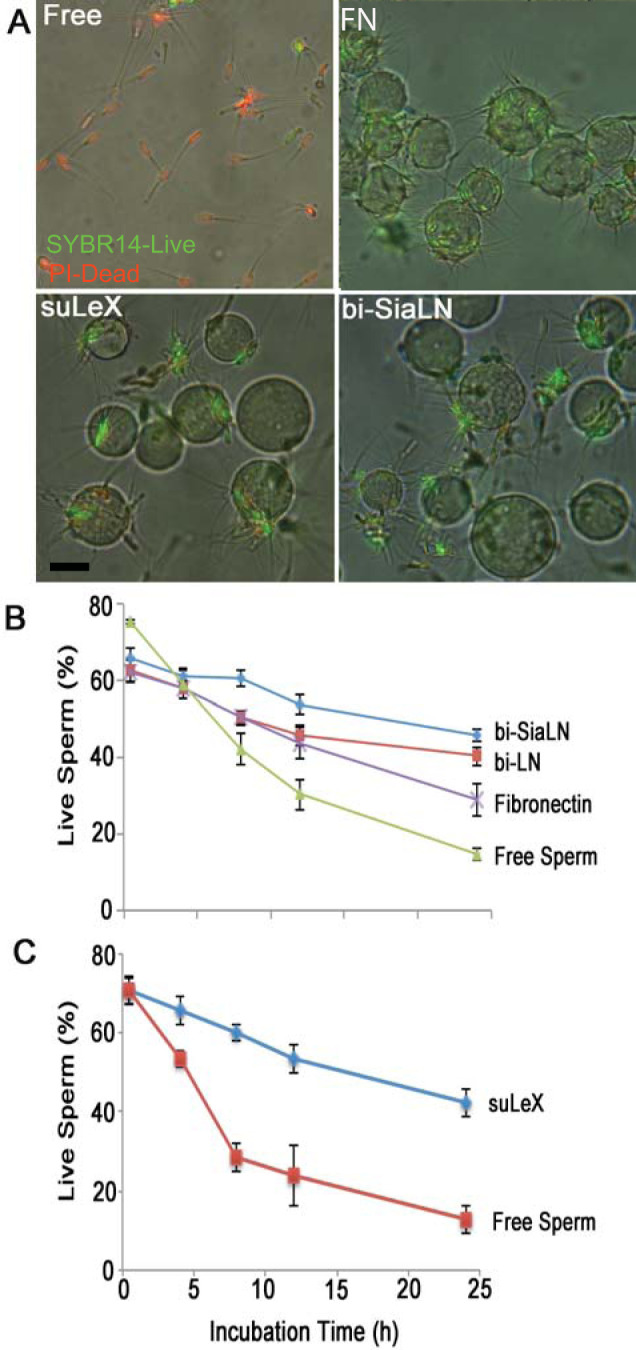
Sperm binding to immobilized bi-SiaLN and suLe^X^ glycans lengthens lifespan. Sperm were incubated with biantennary 6-sialylated *N*-acetyllactosamine oligosaccharide (bi-SiaLN), biantennary *N*-acetyllactosamine terminating oligosaccharide (bi-LN), 3-O-sulfated Lewis X trisaccharide (suLe^X^), 3-O-sulfated Lewis A trisaccharide (suLe^A^), or fibronectin (FN) coupled to beads. Free-swimming sperm were used as controls. ***A***) Representative photos of sperm stained with SYBR14 (green, live) and propidium iodide (red, dead) after 8 hr of incubation showing free-swimming sperm in medium, sperm bound to suLe^X^, bi-SiaLN, or fibronectin (FN) coupled to beads. Scale bar in lower left panel indicates 20 μm. ***B***) Enumeration of the percentage of live (SYBR14^+^, propidium iodide^-^) free-swimming sperm or sperm bound to bi-SiaLN, bi-LN, or FN on beads. ***C***) Sperm incubated with suLe^X^ coupled to beads. Sperm that were bound to biantennary glycans with or without sialic acid residues on *N*-acetyllactosamine or bound to suLe^X^ maintained higher viability than free-swimming sperm and sperm bound to FN-coated beads. The asterisks represent significant differences from free-swimming sperm (*P* < 0.05). These results are means and SEM from 3–5 experiments.

**Fig 3:**
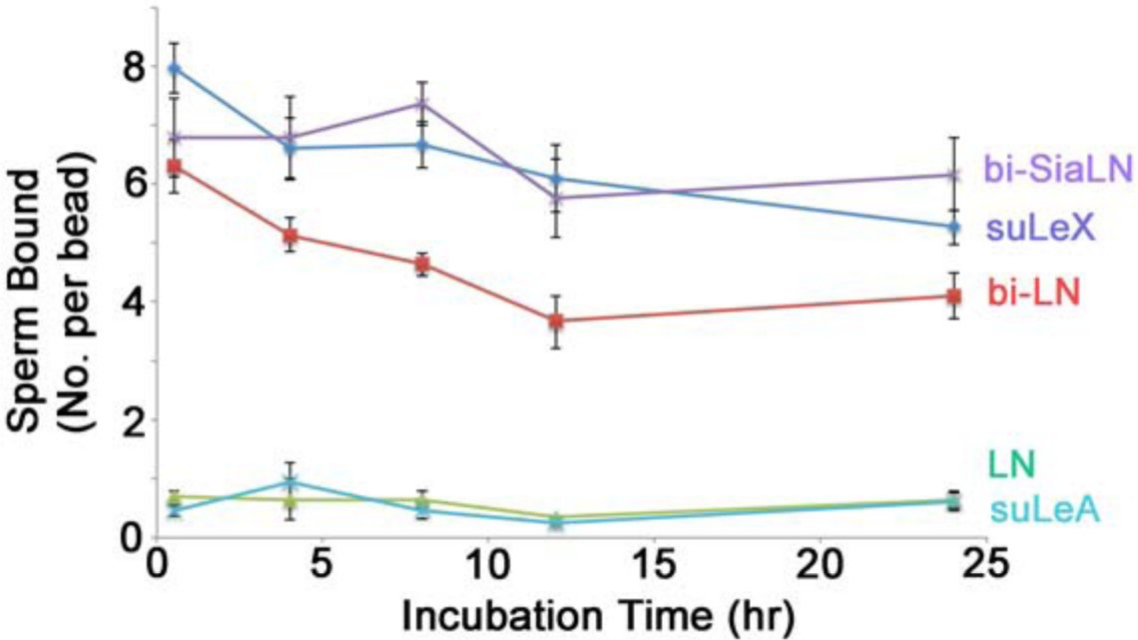
Most sperm remained bound to immobilized oviduct glycans for 24 h. Sperm were incubated with beads coupled with biantennary 6-sialylated *N*-acetyllactosamine oligosaccharide (bi-SiaLN), biantennary *N*-acetyllactosamine terminating oligosaccharide (bi-LN), 3-O-sulfated Lewis X trisaccharide (suLe^X^), 3-O-sulfated Lewis A trisaccharide (suLe^A^), or *N*-acetyllactosamine (LN). The number of bound sperm per bead was counted at time points over 24 h. There was a slight decline in the number of bound sperm only in the sperm bound to bi-LN coated beads (*P*<0.05). Therefore, the number of live sperm bound to the beads was not affected significantly by a release of moribund sperm from the beads.

Sperm storage in the oviductal isthmus delays sperm capacitation and lengthens sperm lifespan, increasing the opportunity for ovulated oocytes to be fertilized [14,38]. Sperm bind to specific glycans on the isthmic epithelium but whether glycan-binding alone could lengthen sperm lifespan was unknown. We tested the direct influence of individual oviduct glycans that bind porcine sperm, bi-SiaLN and Le^X^ structures, and related controls (Fig 1) on sperm viability over 24 h under capacitating conditions (e.g., dmTALP containing BSA, Ca^2+^, and HCO_3_^-^). We used glycans attached to a 30-kDa polyacrylamide chain, so-called neoglycoproteins, to mimic the multivalency common in glycoproteins. These glycans were attached to agarose beads to make them insoluble, resembling their attachment to oviduct epithelial cells. Following incubation of sperm with beads and gentle washing, some sperm remained bound to bi-SiaLN, suLe^X^ and fibronectin (FN) bound to beads but not to LN-beads, a disaccharide found in both suLe^X^ and bi-SiaLN and used as a control (Fig 2A, LN not shown). Binding to bi-SiaLN increased the percentage of viable sperm 2 to 3.5-fold from 8 to 24 h, when compared to free-swimming sperm (Fig 2B, *P*<0.05). Although not as potent as bi-SiaLN, a biantennary oligosaccharide with *N*-acetyllactosamine termini (bi-LN; same as bi-SiaLN except lacking sialyl residues), which binds fewer sperm [43], still induced a 1.7, 1.9, and 3.2 fold increase in viability at 8, 12, and 24 h, respectively, compared to free sperm (*P*<0.05). Sulfated suLe^X^ also extended sperm lifespan during 4–24 h incubation *in vitro* (Fig 2C). Compared to free-swimming sperm, suLe^X^ promoted a 1.3, 2, 3.2, and 4.4-fold increase in viability at 4, 8, 12, and 24 h, respectively (*P*<0.05). The increase in viability was not due only to tethering of a sperm because when fibronectin was linked to the agarose beads and then incubated with sperm, although a high number of sperm bound to the beads, fibronectin did not increase sperm lifespan to the same degree as glycan-bound beads (Fig 2A; *P*<0.05). The increase in viability was not because moribund sperm were released from the glyco-beads. The number of sperm bound to any of the glyco-beads did not change significantly over 24 h, demonstrating that the percentage of viable sperm was not affected by the release of moribund sperm (Fig 3). Therefore, oviduct glycans that bound sperm prolonged sperm lifespan.

### Sperm binding to bi-SiaLN and Le^X^ suppresses Ca^2+^ influx

**Fig 4:**
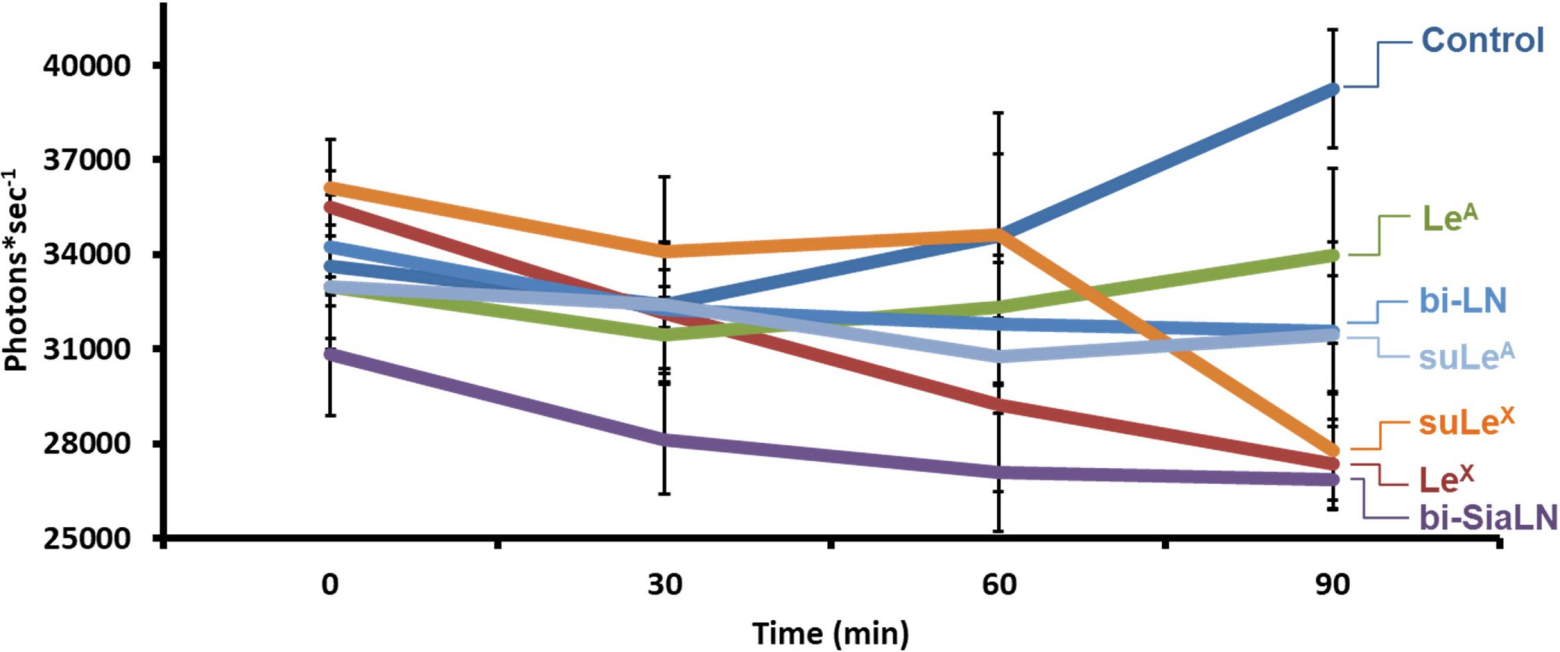
Soluble bi-SiaLN and Le^X^ glycans suppress intracellular Ca^2+^ increase during porcine sperm capacitation. Fluo-4 loaded sperm were incubated with biantennary 6-sialylated *N*-acetyllactosamine oligosaccharide (bi-SiaLN), 3-O-sulfated Lewis X trisaccharide (suLe^X^), 3-O-sulfated Lewis A trisaccharide (suLe^A^), Le^X^, Le^A^, and *N*-acetyllactosamine (LN) and spectrofluorometric readings were taken for 90 min. The intensity of the fluorescent signal (photons per second) is related in intracellular Ca^2+^ concentration. Sperm incubated with bi-SiaLN, Le^X^ and suLe^X^ displayed a suppression in the Ca^2+^ influx that normally accompanies capacitation, as seen in the control group. Statistical differences (*P* < 0.05) compared to the control are labelled by an asterisk. These results are means and SEM from 3–5 experiments.

Previous studies indicated that binding to the oviduct regulated sperm intracellular Ca^2+^ [20,31]. Limiting Ca^2+^ entry into sperm may lengthen sperm lifespan by delaying capacitation [39]. Using spectrophotometry and a semi-quantitative analysis, we determined if oviduct glycan binding influenced intracellular free Ca^2+^ in sperm using Fluo-4, a Ca^2+^-sensitive reporter. Because sperm bound to agarose beads would pull sperm to the bottom of the cuvette quickly, we used neoglycoproteins (glycans linked to the 30 kDa soluble polyacrylamide chain) for this experiment. Although there was no difference in the first 60 min, control sperm showed a gradual, albeit non-significant, increase in intracellular free Ca^2+^ (*P*>0.10). By 90 min of incubation, glycans that bound sperm, bi-SiaLN, Le^X^, and suLe^X^ were able to suppress completely the increase in intracellular free Ca^2+^ in sperm (Fig 4; *P*<0.05). In fact, in the presence of bi-SiaLN, Le^X^, and suLe^X^, there was less intracellular Ca^2+^ at 90 min than at 0 min. The presence of biantennary structure and a sialyl residue attached to the 6-position of each galactosyl residue in bi-SiaLN, which are required for maximum sperm binding [43], were also necessary for the delay in the Ca^2+^ increase because the disaccharide, *N*-acetyllactosamine, did not affect Ca^2+^ (*P*>0.10). Furthermore, the positional isomers of Le^X^ and suLe^X^, Lewis A (Galβ1-3(Fucα1–4)GlcNAc) and sulfated Lewis A (3’-(O-SO_3_)Galβ1-3(Fucα1–4)GlcNAc) had no effect on intracellular Ca^2+^ (*P*>0.10). Thus, very specific structures were required for glycans to affect intracellular Ca^2+^.

### Assessing capacitation by evaluating sperm motility patterns, protein tyrosine phosphorylation, and induced acrosome reaction

Intracellular free Ca^2+^ influences many sperm behaviors such as the development of hyperactivated motility, sperm protein tyrosine phosphorylation and the ability to response to acrosome reaction inducers [51]. CASA observation of sperm motility parameters incubated with soluble oviduct glycans showed no changes when compared with controls (Table 1). Binding to oviduct glycans did not delay hyperactivation in sperm or affect any sperm kinematics.

**Table 1:** Sperm motility parameters after 4 h incubation with soluble suLe^X^, bi-SiaLN, or without glycans.

Motility Parameters after 4 h	C	NC	suLe^X^	bi-SiaLN	suLe^A^	LN
Motility %	41.2 ± 7.8	38.8 ± 7.4	41.8 ± 8.6	41.2 ± 8.7	47.6 ± 8.5	44.0 ± 8.6
Progressive cells %	21.0 ± 5.6	14.2 ± 3.2	16.4 ± 3.8	18.6 ± 4.9	22.8 ± 4.8	15.6 ± 3.5
Rapid cells %	29.6 ± 5.7	24.7 ± 4.5	26.6 ± 5.3	27.8 ± 6.5	33.2 ± 6.0	29.4 ± 6.6
(VAP) um/sec	68.7 ± 3.3	57.0 ± 5.1	66.1 ± 6.3	69.0 ± 5.3	68.3 ± 1.8	65.2 ± 4.8
(VSL) um/sec	40.6 ± 3.1	31.3 ± 2.9	36.5 ± 2.6	39.7 ± 3.5	41.4 ± 1.45	37.3 ± 3.9
(VCL) um/sec	150.2 ± 6.7	128.8 ± 9.4	151.8 ± 3.2	155.1 ± 9.6	150.4 ± 3.2	145.8 ± 8.3
(ALH) um	7.9 ± 0.18	7.5 ± 0.4	8.4 ± 0.3	8.5 ± 0.2	8.0 ± 0.27	7.6 ± 0.2
(BCF) Hz	37.6 ± 0.9	37.0 ± 1.7	38.8 ± 0.6	36.0 ± 0.8	37.1 ± 0.4	37.6 ± 0.8
Straightness %	56.6 ± 3.5	54.4 ± 1.9	53.8 ± 2.5	54.8 ± 2.3	57.8 ± 2.7	54.2 ± 2.8
Linearity %	27.8 ± 1.4	25.0 ± 0.5	24.8 ± 1.1	26.0 ± 1.6	31.0 ± 3.1	29.4 ± 3.1
Static cells %	50.0 ± 8.1	54.6 ± 8.3	46.4 ± 10.0	49.6 ± 9.1	43.4 ± 8.2	47.4 ± 8.7

**Fig 5:**
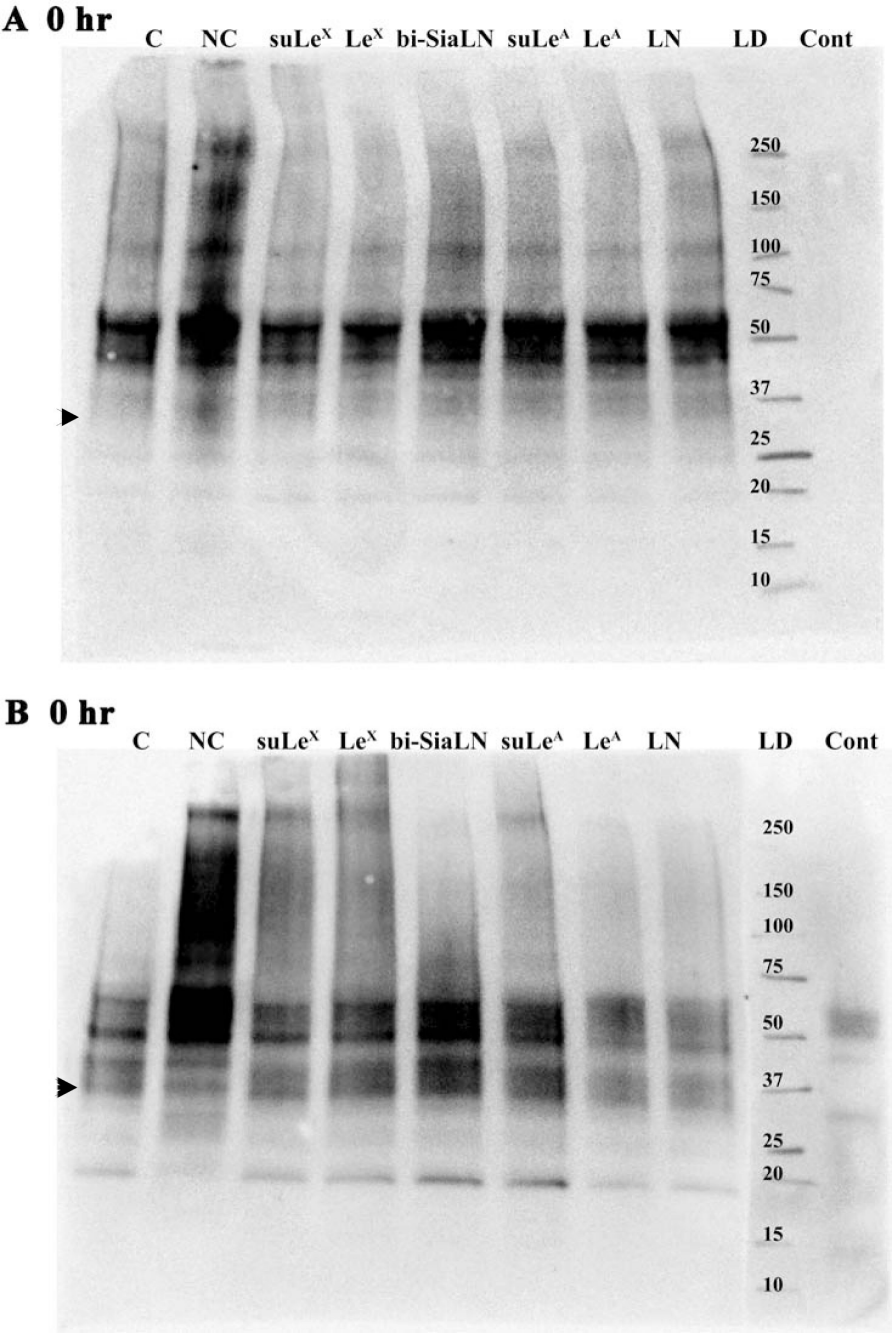
Sperm protein tyrosine phosphorylation increases during capacitation but is not affected by oviduct glycans. SDS-PAGE and immunoblotting with a monoclonal phosphotyrosine antibody (4G10) to detect specific phosphotyrosine-containing proteins showed no differences in the sperm incubated with soluble oviduct glycans when compared to controls at ***A****)* 0 h or ***B****)* 4 h of incubation for capacitation. An arrow indicates the migration of sp32, a sperm protein that is phosphorylated on tyrosine residues as sperm are incubated in capacitating conditions. Capacitating dmTALP (C), non-capacitating dmTALP (NC), C with 3-O-sulfated Lewis X trisaccharide (suLe^X^), C with biantennary 6-sialylated *N*-acetyllactosamine oligosaccharide (bi-SiaLN), C with 3-O-sulfated Lewis A trisaccharide (suLe^A^), C with *N*-acetyllactosamine disaccharide (LN), Ladder (LD) and control without primary antibody (Cont), n = 3.

Tyrosine phosphorylation of a 32 kDa sperm protein was used as an assessment of capacitation [52]. Phosphorylation of sp32 in porcine sperm is increased during a 4.5 h capacitation time, although sp32 phosphorylation is not completely diagnostic of capacitation because it increases in medium without HCO_3_^-^ that does not capacitate sperm [53]. Tyrosine phosphorylation of sp32 was increased after a 4 hr incubation of sperm in capacitating conditions and also in the absence of BSA and HCO_3_^-^ (Fig 5), in agreement with previous results [53]. This increase was not affected by addition by either of the soluble oviduct glycans or controls.

**Fig 6:**
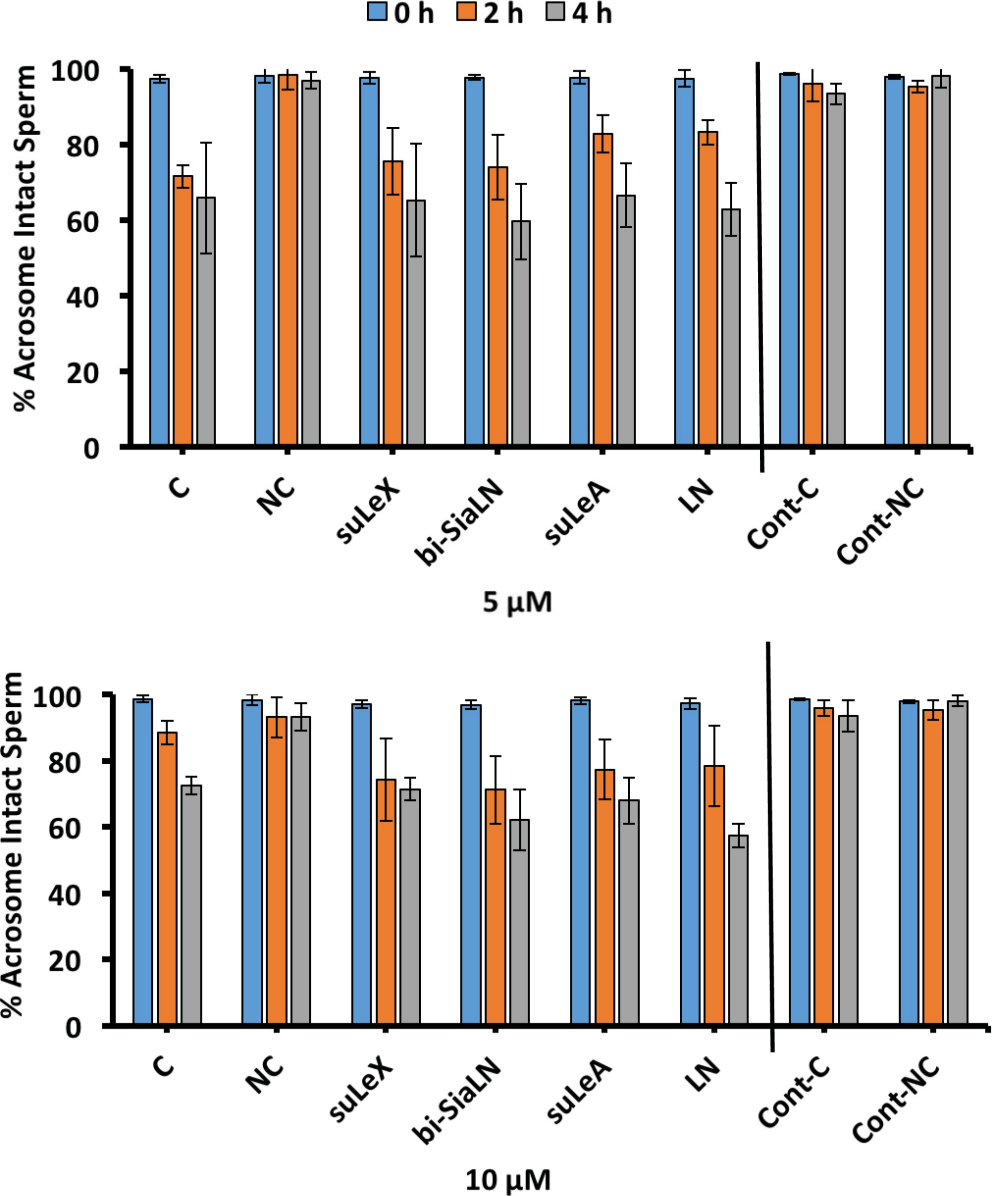
Oviduct glycans do not affect capacitation, as assessed by the ionophore-induced acrosome reaction. Sperm were incubated in capacitating medium dmTALP (C), non-capacitating medium NC-TALP (NC), C with 3-O-sulfated Lewis X trisaccharide (suLe^X^), C with biantennary 6-sialylated *N*-acetyllactosamine oligosaccharide (bi-SiaLN), C with 3-O-sulfated Lewis A trisaccharide (suLe^A^), C with *N*-acetyllactosamine disaccharide (LN), all with A23187, C without A23187 (Cont-C) and NC without A23187 (Cont-NC). The acrosome reaction was induced by two concentrations of Ca^2+^ ionophore (A23187) after 0, 2 and 4 h of capacitation. ***A*)** 5 μM A23187. ***B*)** 10 μM A23187. Although A23187 induced loss of sperm acrosomes, neither oviduct glycan changed the frequency of A23187-induced acrosome reactions, graphed as a loss of acrosome-intact sperm. These results are means and SEM from 3–5 experiments.

As the acrosome reaction requires sperm to be capacitated, we expected that if oviduct glycans delayed capacitation, a delay in the induced acrosome reaction (a decrease in intact acrosomes) would follow, as previously published in porcine sperm [53]. Addition of either 5 μM or 10 μM of Ca^2+^ ionophore (A23187) decreased the number of sperm with intact acrosomes when added at either 2 h or 4 h (Fig 6A and 6B, respectively). When soluble glycans were added to sperm during the entire incubation, the addition of A23187 at 2 h and 4 h of incubations, oviduct glycans did not affect the acrosome reaction induced by either concentration of A23187) (Fig 6).

Sperm were incubated in capacitating dmTALP (C), NC-TALP (NC), C with 3-O-sulfated Lewis X trisaccharide (suLe^X^), C with biantennary 6-sialylated *N*-acetyllactosamine oligosaccharide (bi-SiaLN), C with 3-O-sulfated Lewis A trisaccharide (suLe^A^), and C with *N*-acetyllactosamine (LN). Path velocity (VAP) μm/sec, progressive velocity (VSL) μm/sec, track speed (VCL) μm/sec, amplitude of lateral head displacement (ALH) μm, beat cross frequency (BCF) Hz. Results are means and SEM from 3–5 experiments.

## Discussion

Formation of a sperm reservoir *in vivo* requires sperm binding to oviductal cells of the lower oviduct or isthmus. Binding to oviductal cells lengthens sperm lifespan and suppresses Ca^2+^ influx [39,40]. Previously, the oviduct cell components that mediated these functional outcomes on sperm were unknown. It was also not clear whether the sperm-oviduct adhesive molecules might have additional roles in regulating sperm function. The current study showed that oviduct glycan motifs on the luminal epithelium of the isthmus, both bi-SiaLN and suLe^X^, regulate sperm intracellular Ca^2+^ concentration and lengthen sperm lifespan. In contrast, we found no evidence that binding to oviduct glycans affects sperm capacitation or development of hyperactivated motility. Nevertheless, adhesion to oviduct glycans is responsible for at least two outcomes of sperm interaction with oviduct cells.

The effect of glycan binding on the suppression of Ca^2+^ influx was very specific. Under normal capacitating conditions *in vitro*, the gradual rise in cytosolic Ca^2+^, an altered pattern of flagellar beating and plasma membrane destabilization occur in preparation for the acrosome reaction [54]. Here, we show that at the end of 90-min capacitation time, bi-SiaLN and Le^X^ (sulfated and non-sulfated forms) not only blocked the normal Ca^2+^ influx associated with capacitation but actually reduced intracellular Ca^2+^ below the concentration found at the beginning of capacitation. In contrast, Le^A^, a positional isomer of Le^X^, and LN, a disaccharide that lacks N-acetylneuraminic acid and is not multivalent, did not affect Ca^2+^ entry. These glycans that did not affect intracellular Ca^2+^ also do not bind sperm [42,43]. Regulation of intracellular Ca^2+^ by the oviduct is key to fertility, particularly in species in which fertilization and ovulation are poorly synchronized [13,19]. Binding to oviduct glycans may prevent premature capacitation. Once capacitated, unless the sperm is near an egg, its fate is death; fertility is maximized if capacitation is completed near the time a sperm encounters the egg [14,51,54]. Our data demonstrate that sperm binding to glycans on the epithelium of the oviduct isthmus controls the influx of Ca^2+^, which we propose allows proper timing of sperm capacitation for maximal fertility [23,31,55]. However, we did not observe an effect of oviduct glycans on hyperactivated motility or two other measures of capacitation, sperm sp32 tyrosine phosphorylation or the ability to undergo an induced acrosome reaction.

Although tyrosine phosphorylation of most proteins found in porcine sperm is unchanged during capacitation, phosphorylation of sp32 is increased during capacitation time if extracellular Ca^2+^ is present [53]. This increase in sp32 tyrosine phosphorylation was reported to occur in the absence of NaHCO_3_ and BSA [53] and our data were consistent with that result because sp32 tyrosine phosphorylation increased during incubation time in medium lacking NaHCO_3_ and BSA but with 2 mM Ca^2+^ (Fig 5). Notwithstanding that increased tyrosine phosphorylation does not necessarily demonstrate that capacitation has been completed, because extracellular Ca^2+^ is required for increased sp32 phosphorylation, we anticipated that if the increase in intracellular Ca^2+^ were suppressed, sp32 would be diminished. But sp32 tyrosine phosphorylation was not affected by oviduct glycans. Similarly, soluble oviduct glycans did not affect motility, as evaluated by CASA. The development of hyperactivated motility, as observed by changes in VCL, VSL, VAP, linearity, straightness, ALH, and BCF, is associated with capacitation [56,57]. However, these motility characteristics were unchanged by soluble glycans. Furthermore, the ability of sperm to respond to A23187 with acrosome reactions, a measure used to assess capacitation of porcine sperm [53], was not affected by addition of soluble oviduct glycans. One possible explanation is that the oviduct glycans must be immobilized by cells or beads to affect capacitation; however, soluble glycans suppressed the increase in intracellular Ca^2+^. An alternative possibility is that capacitation includes many different processes including some that are independent of each other, and those we examined happen to be among those that were unchanged. Regardless, the assessments of capacitation we used, which are common assessments, were not affected by soluble glycans.

Direct membrane contact between spermatozoa and epithelial cells of the isthmus is necessary to regulate Ca^2+^ entry, capacitation, minimize oxidative damage and maintain sperm viability over extended periods and improving in vitro fertilization success of sperm released from oviduct cells [20,38,39,58]. The carbohydrate interactions studied in this work are at least partially responsible for sperm adhesion to the oviduct and indicate that bi-SiaLN and Le^X^ each regulate porcine sperm viability. Viability was tested over a 24 h-period using agarose beads coated with specific glycans as binding matrices. The effect of glycan binding on sperm viability was clear as soon as 4 and 8 h of incubation with suLe^X^ and bi-SiaLN coated beads, respectively. The prolonged viability may be due to suppression of the Ca^2+^ influx. Sperm binding to the isthmic epithelium delays capacitation [38] and lengthens sperm fertilizing ability [40], perhaps by suppressing the Ca^2+^ influx associated with capacitation. The time course of the Ca^2+^ measurements was much different than the viability measurements. Due to leakage of Fluo-4 from the cells, we were unable to make accurate measurements of intracellular Ca^2+^ over longer time intervals. But the glycans that suppressed Ca^2+^ influx at 90 min were also the glycans that extended sperm lifespan as late as 24 h.

The increase in sperm lifespan had two components. The first component was an increase in sperm lifespan by simply anchoring sperm to a bead, exemplified by sperm binding to fibronectin-coated beads. There was a significant increase in viability compared to free-swimming sperm. The second component was glycan dependent. An even higher percentage of sperm bound to beads coated with bi-SiaLN and Le^X^ were alive after up to 24 h. Although immobilizing sperm on fibronectin or glycan coated beads improved their lifespan (Fig 2), glycans that were not immobilized were still able to suppress the increase in Ca^2+^ (Fig 4). Thus, soluble glycans have the ability to regulate sperm function.

One hypothesis to explain the increased lifespan in glycan-bound sperm is that some unidentified growth factors that lengthen sperm lifespan might be bound to the glycans in the same way that growth factors bind and are stabilized by proteoglycans [59]. However, the glycans we used are not related to glycosaminoglycans, the glycans commonly found on proteoglycans. Furthermore, prior to binding to beads, sperm are washed extensively in a medium lacking protein except BSA. Thus, a direct effect of the glycan ligand binding to unknown receptors seems more likely. A rational exploration of the biological effects of binding to glycan receptors awaits identification of these receptors.

This study shows that oviduct Le^X^ and bi-SiaLN glycans suppressed the influx of Ca^2+^and extended sperm viability. This suggests that binding to specific components of the extracellular matrix can lengthen the lifespan of a cell normally in suspension, like sperm. Although there is much information about how the extracellular matrix affects cell behavior [27,60], to our knowledge, these data are the first to demonstrate that adhesion specifically to a glycan matrix can affect the viability of sperm or any other cells and can influence intracellular Ca^2+^.

## Supporting information

S1 Fig The original blot used for Fig 5. (PDF)

S1 Data An excel file with the raw data from Figs 2, 3, 4, and 6 is available. (XLSX)
