# Supplementary material for: Adhesion to oviduct glycans regulates porcine sperm Ca2+ influx and viability
Source: PLoS One. 2020 Aug 21;15(8):e0237666. doi: 10.1371/journal.pone.0237666 (PMC7442259; doi:10.1371/journal.pone.0237666)
Supplement: S1 Fig — (PDF) [file pone.0237666.s001.pdf]

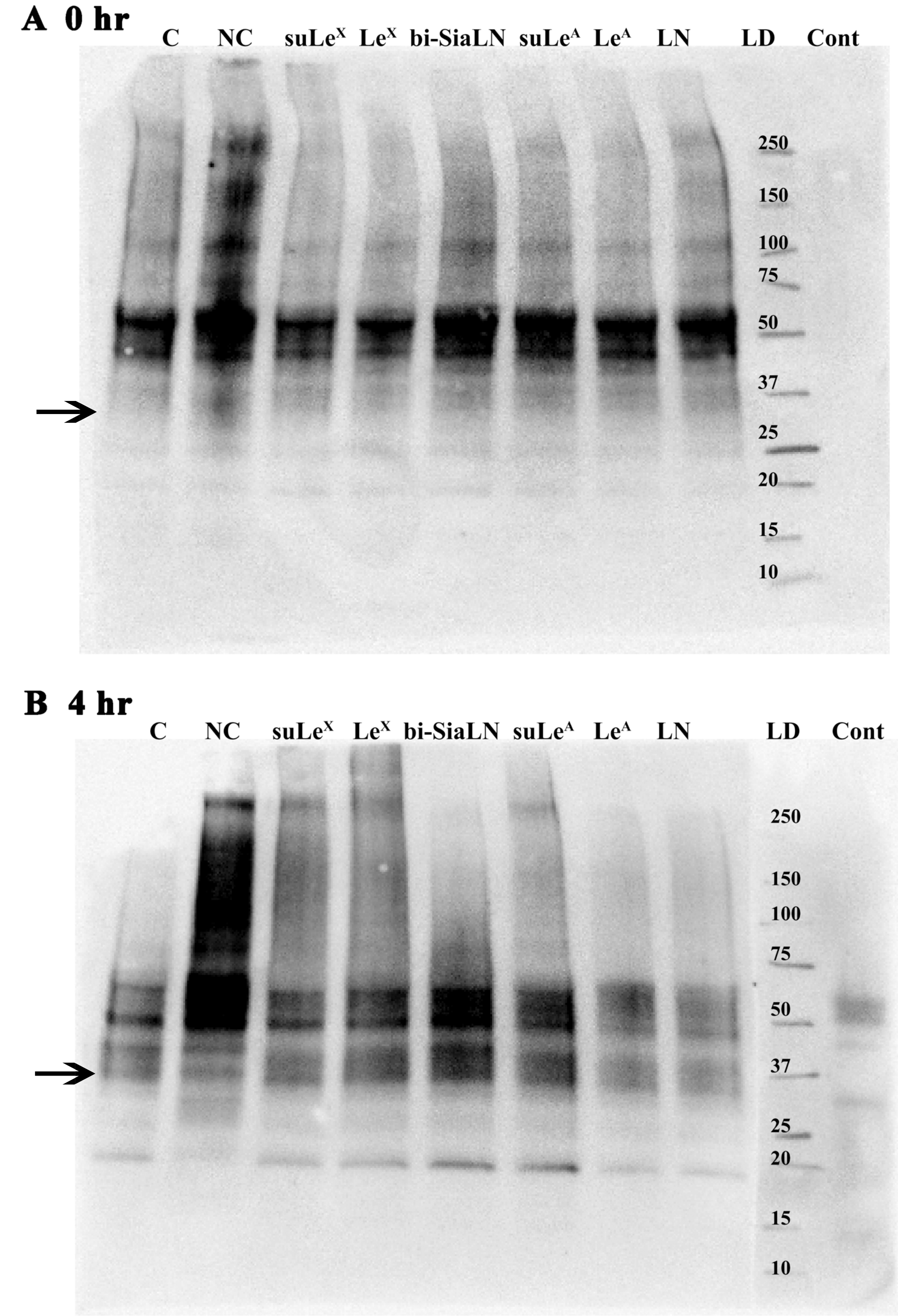
A 0 hr
C
NC
suLeX
LeX
bi-SiaLN
suLeA
LeA
LN
LD
Cont
250
150
100
75
50
37
25
20
15
10
B 4 hr
C
NC
suLeX
LeX
bi-SiaLN
suLeA
LeA
LN
LD
Cont
250
150
100
75
50
37
25
20
15
10
